# Supplementary material for: Stable integration of an optimized inducible promoter system enables spatiotemporal control of gene expression throughout avian development
Source: Biol Open. 2020 Oct 6;9(10):bio055343. doi: 10.1242/bio.055343 (PMC7561481; doi:10.1242/bio.055343)
Supplement: Supplementary information [file biolopen-9-055343-s1.pdf]

Table S1

qPCR primers	
CXCL14 F3 Chick	5’-GCAGAAGGAGTAAAGTGCAA-3’
CXCL14 R3 Chick	5’-GTACCACTTGAGCAGCCTCA-3’
Runx2 F1 CQ DC	5’-CCGTCCTACTTGAGCCAGAT-3’
Runx2 R1 CQ DC	5’-ACGTCGGTGATGGCTGGAAG-3’
MMP13_F1_universal_SS	5’-CCTGATGATGATGTGCAAG-3’
MMP13_R1_universal_SS	5’-CCTGTCCTTGAAGACCAG-3’
Gas1_F1_Chick_Quail_ZV	5’-CCGCTACATGGCCTACTG-3’
Gas1_R2_Chick_ZV	5’-CTTGACCGACTCGCAGAT-3’

Cloning primers	
GAS1 FL F1 CQ DC	5’-TGGATTGATGCGAGGAGACC-3’
GAS1 FL R1 CQ DC	5’-CACACGGGGACAGACACAC-3’
pTet GAS1 F1 CQ DC	5’-ACCCTCGTAAAGCCGCCACCATGCCGGCCCGCCCG-3’
pTet GAS1 R1 CQ DC	5’-GCCGCTTCACTTGTACTGCACTAGAGCGGCGGTAGCAGC-3’
CXCL14 5UTR C F1 DC	5’-GAACACAAGACAGAACCCCG-3’
CXCL14 3UTR U R1 DC	5’-GGTGTGAAATCTGAAGTGCA-3’
pTet Cxcl14 F1 U DC	5’-ACCCTCGTAAAGCCGCCACCATGAAGCTCCTGACAGC-3’
pTet Cxcl14 R1 CQ DC	5’-GCCGCTTCACTTGTACTGCACGCCGCCTCCTATTCTTCAT-3’
Mmp13 C F1 FL DC	5’-ATGCAACCCAGACTTTCAGC-3’
Mmp13 C R1 FL DC	5’-GGTAGTCAGTGCTTGTTCGC-3’
pTet MMP13 F1 C DC	5’-ACCCTCGTAAAGCCGCCACCATGCAACCCAGACTTTCAGC-3’
pTet MMP13 R1 CQ DC	5’-GCCGCTTCACTTGTACTGCATCAGCACCAAAATAAGGAGT-3’
Runx2 C F2 FL DC	5’-ATGGCATCAAACAGCCTCTT-3’
Runx2 U R1 FL DC	5’-TCAGTACGGCCTCCAAACG-3’
pTET Runx2 F1 CQ DC	5’-ACCCTCGTAAAGCCGCCACCATGGCATCAAACAGCCTCTT-3’
pTET Runx2 R1 U DC	5’-GCCGCTTCACTTGTACTGCATCAGTACGGCCTCCAAACG-3’
pTet H2B F1 DC	5’-GGCCTTTCGGCCGCCACCATGCCAGAGCCAGCGAAG-3’
pTet H2B R1 DC	5’-CCTCCTCGCCCTTGCTCACCATGGTGGCGACCGGTGGAAC-3’
pNanoPB mPGK R2 DC	5’-GGTGGCGGCCGAAAGGCCCGGAGATGAGG-3’
pPID2 Tet Bi F1 DC	5’-GGAGCGACAGTGGTAGACAGCCCCATAGAGCCC-3’
Fluor Tet Bi F1 DC	5’-ATGGTGAGCAAGGGCGAGG-3’
rbGlob Tet Bi R1 DC	5’-TTAAGAGTCGTCACCTGCAGTACAAGTGAAGCGGCCGGCC-3’
Tet rbGlob F1 DC	5’-CTGCAGGTGACGACTCTTAAGGTGGCGGCTTTACGAGGGTAGGAAGTGG-3’
Ori F1 DC	5’-GCCTCACTGATTAAGCATTGGTACCCGTAGAAAAGATCAAAGGATC-3’
Ori R1 DC	5’-ACATATTTCTCGAGATATCGAATTCGTTTTTCCATAGGCTCCGCC-3’
BlaR F1 DC	5’-GGAAAAACGAATTCGATATCTCGAGAAATATGTATCCGCTCATGAGAC-3’
BlaR R1 DC	5’-GATCCTTTGATCTTTTCTACGGGTACCAATGCTTAATCAGTGAGGC-3’
pNano PB 5'LTR R DC	5’-GGCTGTCCCTGATATCTATAACA-3’

Biology Open • Supplementary information

pNanoPB mPGK F1 DC	5'-TATAGATATCAGGGACAGCCGGGTAGGGGAGGCGCTTT-3'
pNanoPB mPGK R2 DC	5'-GGTGGCGGCCGAAAGGCCCGGAGATGAGG-3'
pNano rbGlob R1 DC	5'-TCATGAGCGGATACATATTTGAGAAGAGGGACAGCTATGA-3'
pNano PB 3'LTR F2 DC	5'-GGCTGTCCCTCATAAAAGTTTTGTTACTTTATAGA-3'
pNanoPB rbGlob R1 DC	5'-AACTTTTATGAGGGACAGCCGAGAAGAGGGACAGCTATGA-3'
pNano PB 3'LTR R2 DC	5'-GAGCGGATACATATTTCTCGTTAACCCTAGAAAGATAATC-3'
Fluor F1 DC	5'-ATGGTGAGCAAGGGCGAGG-3'
pNano PB 3'LTR R2 DC	5'-GAGCGGATACATATTTCTCGTTAACCCTAGAAAGATAATC-3'
pNano HyPBase F2 DC	5'-CCTCATCTCCGGGCCTTTCGAATTCGCCGCCACCATGGG-3'
pNano HyPBase R2 DC	5'-CCGGCCGCTTCACTTGTATCAGAAACAGCTCTGGCACA-3'
pNano mPGK F1 DC	5'-GGCGGAGCCTATGGAAAAACGGGTAGGGGAGGCGCTTT-3'
pPID PGK F1 DC	5'-GGAGGGAGCGACAGTGGTGGGTAGGGGAGGCGCTT-3'
pNanoPB rbGlob F1 DC	5'-TACAAGTGAAGCGGCCGG-3'
pE Kozak F1 DC	5'-GCCTTTCGATTGCCGCCACC-3'
pNanoPB IRES F1	5'-CCTCATCTCCGGGCCTTTCGGAATTCGTGGACTCTCGAGG-3'
Tet mNG R1 DC	5'-GCAACTAGAAGGCACAGTCGTTACCCGGGGAGCATGTCAA-3'
Tet mNG F1 DC	5'-TGGAGGAGAACCCCGGCCCCATGTCTAGACTGGACAAGAGC-3'
mNG P2A R1 DC	5'-GGGGCCGGGGTTCTCCTCCA-3'
Tet Bi mNG F1 DC	5'-CCTCATCTCCGGGCCTTTCGGCCGCCACCATGGTGAGCAAGGGCGA-3'
Tet Bi FL F1 DC	5'-GAAAAGCGCCTCCCCTACCCGAGAAGAGGGACAGCTATGA-3'
PGK FL F1 DC	5'-GGGTAGGGGAGGCGCTTTTC-3'
rbGlob Tet Bi R1 DC	5'-TTAAGAGTCGTCACCTGCAGTACAAGTGAAGCGGCCGGCC-3'
Tet rbGlob F1 DC	5'-CTGCAGGTGACGACTCTTAAGGTGGCGGCTTTACGAGGGTAGGAAGTGG-3'
Tet Fluor R2 DC	5'-TCCTCGCCCTTGCTCACCATGGTGGCGGCGGTGAATTCTCCAGGCGATC-3'
BGH Tet Bi F1 DC	5'-CGACTGTGCCTTCTAGTTGC-3'
Fluor Tet Bi R1 DC	5'-GCAACTAGAAGGCACAGTCGTCACTTGTACAGCTCGTCCA-3'
pPID2 Tet Bi R1 DC	5'-GAGAGCACGAGTCATCTAGAGGACAGCCATAGAGCCCA-3'

Biology Open • Supplementary information

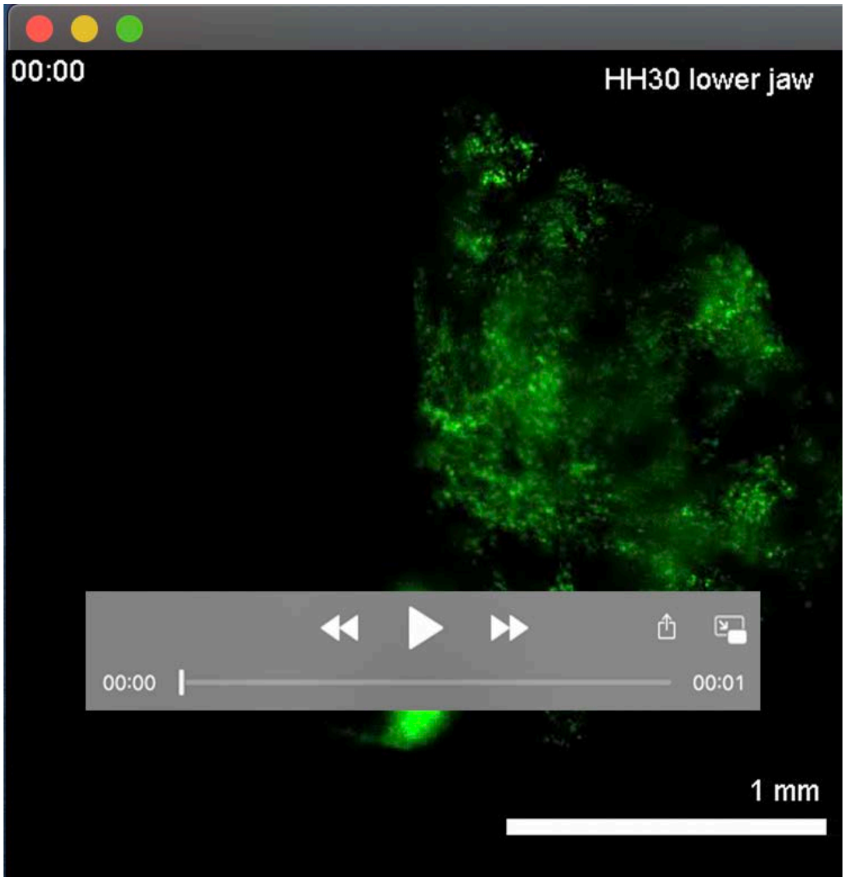

Movie 1

Biology Open • Supplementary information
